# N_2_ Fixation, N Transfer, and Land Equivalent Ratio (LER) in Grain Legume–Wheat Intercropping: Impact of N Supply and Plant Density

**DOI:** 10.3390/plants13070991

**Published:** 2024-03-30

**Authors:** Sebastian Salinas-Roco, Amanda Morales-González, Soledad Espinoza, Ricardo Pérez-Díaz, Basilio Carrasco, Alejandro del Pozo, Ricardo A. Cabeza

**Affiliations:** 1 Laboratory of Plant Nutrition, Department of Crop Sciences, Faculty of Agricultural Sciences, University of Talca, Talca 3460000, Chile; sebastian.salinas@utalca.cl (S.S.-R.); amanda.morales@utalca.cl (A.M.-G.); 2 Centro Regional de Investigación Quilamapu, Instituto de Investigaciones Agropecuarias, Chillán 3780000, Chile; soledad.espinoza@inia.cl; 3 Centro de Estudios en Alimentos Procesados (CEAP), Talca 3480094, Chile; rperez@ceap.cl (R.P.-D.); bcarrasco@ceap.cl (B.C.); 4 Plant Phenomics Center, Faculty of Agricultural Sciences, University of Talca, Talca 3460000, Chile; adelpozo@utalca.cl

**Keywords:** legumes, cereal, intercropping, N_2_ fixation, N transfer

## Abstract

Intercropping legumes with cereals can lead to increased overall yield and optimize the utilization of resources such as water and nutrients, thus enhancing agricultural efficiency. Legumes possess the unique ability to acquire nitrogen (N) through both N_2_ fixation and from the available N in the soil. However, soil N can diminish the N_2_ fixation capacity of legumes. It is postulated that in intercropping, legumes uptake N mainly through N_2_ fixation, leaving more soil N available for cereals. The latter, in turn, has larger root systems, allowing it to explore greater soil volume and absorb more N, mitigating its adverse effects on N_2_ fixation in legumes. The goal of this study was to evaluate how the supply of N affects the intercropping of faba beans (*Vicia faba* L.) and peas (*Pisum sativum* L.) with wheat under varying plant densities and N levels. We measured photosynthetic traits, biomass production, the proportion of N derived from air (%Ndfa) in the shoot of the legumes, the N transferred to the wheat, and the land equivalent ratio (LER). The results revealed a positive correlation between soil N levels and the CO_2_ assimilation rate (An), chlorophyll content, and N balance index (NBI) in wheat. However, no significant effect was observed in legumes as soil N levels increased. Transpiration (E) increased in wheat intercropped with legumes, while stomatal conductance (g_s_) increased with N addition in all crops. Water use efficiency (WUE) decreased in faba beans intercropped with wheat as N increased, but it showed no significant change in wheat or peas. The shoot dry matter of wheat increased with the addition of N; however, the two legume species showed no significant changes. N addition reduced the %Ndfa of both legume species, especially in monoculture, with peas being more sensitive than faba beans. The intercropping of wheat alleviated N_2_ fixation inhibition, especially at high wheat density and increased N transfer to wheat, particularly with peas. The LER was higher in the intercropping treatments, especially under limited N conditions. It is concluded that in the intercropping of wheat with legumes, the N_2_ fixation inhibition caused by soil N is effectively reduced, as well as there being a significant N transfer from the legume to the wheat, with both process contributing to increase LER.

## 1. Introduction

Legumes in symbiosis with rhizobia have the unique ability to fix di-nitrogen (N_2_) from the atmosphere for their own metabolic processes. Thanks to this, the use of legumes in agricultural systems reduces the negative impact on the agroecosystem caused by the use of synthetic nitrogen (N) fertilizers [1,2]. Furthermore, legumes, and especially pulses, are a valuable source of protein for human nutrition [3,4] and animal feed [5]. Legumes can also be a good alternative to use in combination with other crops (intercrop) to take advantage of complementary processes [6,7]. This is because growth-promoting resources, such as light, water, and nutrients, are used more efficiently in intercropping [8,9], leading to higher productivity compared to monocrop systems [10]. Furthermore, the N_2_ fixation capacity of legumes is significantly enhanced in intercropping systems with other species, thus increasing soil N levels and improved yields of the companion crops, particularly cereals [11,12,13,14].

Indeed, early studies of peas (*Pisum sativum* L.)–barley (*Hordeum vulgare* L.) intercropping showed strong competition for soil N by the cereal, leading to a notable increase in the proportion of N derived from air (%Ndfa) in peas compared to the monocrop system [15,16]. Also, in faba beans (*Vicia faba* L.)–barley intercropping, the %Ndfa increased on average from 74 to 92% depending on the cropping intensity [17]. Furthermore, the meta-analysis performed by Rodriguez et al. [18], showed that the proportion of %Ndfa increased in average from 66 to 76%, in various grain legumes intercropped with cereals compared to legumes cropped on their own. Apparently, the rise in the %Ndfa in legumes cultivated in intercropping systems correlates with the decline in available soil N, which is attributed to an increased N uptake by the cereal, which subsequently diminishes the inhibition of N_2_ fixation [11,16,19,20].

In legume–cereal intercropping, the latter take advantage of the N transfer facilitated by the N_2_ fixation process carried out by legumes [6]. This N transfer is achieved through the release of N compounds by the legume roots, through a process called rhizodeposition, which proceeds in two ways: (1) the senescence, death, and decomposition of roots/nodules; and (2) the exudation of compounds from the roots, which contain assimilable N [6,21]. Another mechanism by which N transfer occurs is through arbuscular mycorrhizal networks, which spread and connect the root system of legumes and cereals, especially under limited N conditions [22,23]. The N transfer is variable, depending on plant growth conditions. For instance, the N transfer from soybeans to sorghum ranged from 32 to 58% [24], while Chu et al. [25], showed that N transfer from peanut to rice in intercrop was between 6.2 and 12.2%, depending on the N applied as fertilizer. Similarly, Gungaabayar et al. [26] reported N transfer in an intercrop of peas with cereals between 17 and 43%.

Therefore, legume–cereal intercropping is an effective strategy to increase the N availability for the cereal and also to promote the N_2_ fixation of the legumes [27,28,29,30]. However, the benefit of N_2_ fixation is affected by N fertilization by reducing the nodule number and their specific activity in legumes cultivated in monoculture [31,32,33]. The study of Xiao et al. [14] in faba bean–wheat (*Triticum aestivum* L.) intercropping showed that the N fertilization can be reduced by 5 to 15%, but still increasing wheat yield by 16 to 30%. But the impact of N fertilization on N_2_ fixation and the nodulation of grain legumes grown in intercropping systems with cereals remains unclear.

Intercropping systems offer the advantage of more efficient land use, measured by the land equivalent ratio (LER). LER compares the yield of intercropped species to the yield of the same species grown in monoculture on the same area [34]. A value of one indicates no difference, while values above one indicate intercropping outperforms monoculture, and values below one indicate lower yields due to competition [35,36]. Studies have shown LER values above one in legume–cereal intercropping systems [37,38]. For instance, corn intercropped with peanuts, soybeans, and mungbeans had LER values of 1.66, 1.60, and 1.48, respectively [39]. Similarly, pea–barley intercropping showed LER values of 1.14 and 1.10, respectively, indicating 14% and 10% more efficiency compared to monoculture, with the intercrop performing better without additional N fertilizer [40].

In the current study, it was hypothesized that the inhibition of N_2_ fixation induced by the presence of N in the soil could potentially be alleviated through intercropping. This hypothesis is based on the premise that N uptake by the cereal would reduce soil N levels, thus promoting N_2_ fixation by the legume. The objectives of this research were to assess the impact of N supply on the intercropping of peas and faba beans with wheat across two wheat plant densities and three N levels, focusing on: leaf gas exchange and pigment content of the three species, biomass production, the %Ndfa in the legumes, the N transfer to the cereal, and the land equivalent ratio (LER) for the intercropping treatments.

## 2. Results

### 2.1. Gas Exchange Parameters

**Figure 1:**
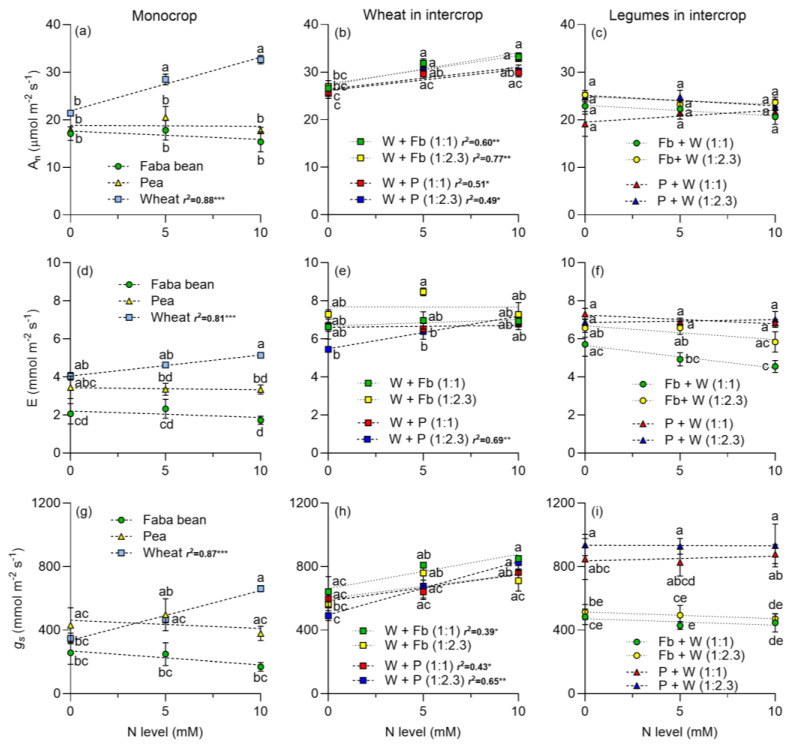
Responses of leaf gas exchange parameters to N supply of faba beans, peas, and wheat growing in monocrop and intercropped at two plant densities: 1:1 and 1:2.3, respectively. A_n_ is the net assimilation of CO_2_ (**a**–**c**); E is the transpiration (**d**–**f**) and g_s_ is the stomatal conductance (**g**–**i**). N levels were zero (N available in the soil), 5, and 10 mM applied in the form of NH_4_NO_3_. Symbols represent the mean and bars the standard error (*n* = 4 for each N level). r^2^ is the coefficient of determination for linear regressions and asterisks indicate statistical significance at *p*-values: * < 0.05, ** < 0.001, and *** < 0.0001, respectively. Small letters indicate differences between treatments according to two-way ANOVA and Tukey tests (*p* < 0.05).

**Figure 2:**
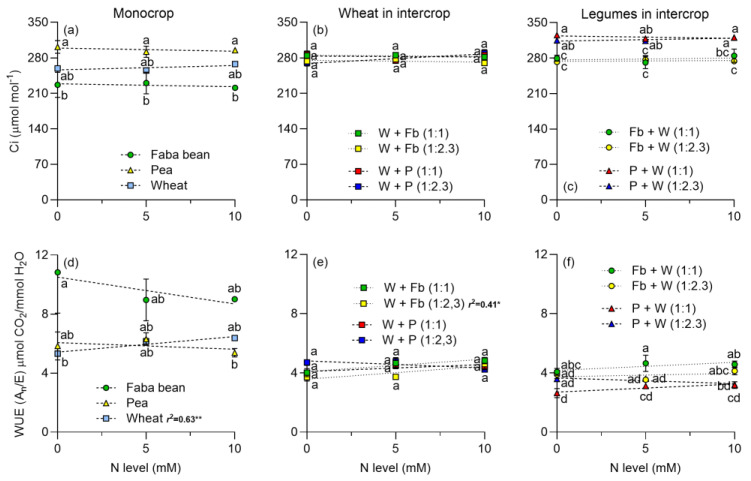
Responses of intercellular carbon (C_i_) and water use efficiency (WUE) to the N level of faba beans, peas, and wheat in monocrop and intercropped at two plant densities: 1:1 and 1:2.3, respectively. C_i_ is the intercellular CO_2_ concentration (**a**–**c**) and WUE is the water use efficiency (**d**–**f**). N levels were zero (N available in the soil), 5, and 10 mM applied in the form of NH_4_NO_3_. Symbols represent the mean and bars the standard error (*n* = 4 for each N level). r^2^ is the coefficient of determination for linear regressions and asterisks indicate statistical significance at *p*-values: * < 0.05, and ** < 0.001, respectively. Small letters indicate differences between treatments according to two-way ANOVA and Tukey tests (*p* < 0.05).

The N level did not affect the net assimilation of CO_2_ (A_n_) of legumes in monocrop or in intercrop with wheat, but had positives effects on wheat growing in monocrop or intercropped with faba beans or peas (Figure 1a,b). A_n_ was slightly higher in legumes intercropped with wheat than legumes cultivated alone (Figure 1c). The plant density had no effects on A_n_ in legumes (Figure 1c). Similarly, the N increased transpiration (E) in wheat (Figure 1d), but not in legumes (Figure 1d). When wheat was intercropped with legumes, E increased, particularly in intercropping with faba beans. For faba beans, E decreased as the N increased and was higher at the plant density of 1:2.3 than at 1:1 (Figure 1f). Conversely, in peas, E nearly doubled at both plant densities compared to sole cultivation, despite N having no effect on E (Figure 1f). The stomatal conductance (g_s_) followed a similar pattern to A_n_; increased in wheat with increasing N level, both in monoculture and when intercropped with legumes (Figure 1g,h), whereas legumes showed no response to N addition (Figure 1g). Interestingly, when intercropped with wheat, g_s_ doubled at both plant densities compared to sole cultivation, despite N level showing no significant effect (Figure 1i). Intercellular CO_2_ concentration (C_i_) remained unchanged with varying N level in both legumes and wheat in monoculture (Figure 2a). Water use efficiency (WUE) exhibited minimal change with N, with a slight increase observed in wheat (Figure 2d).

### 2.2. Photosynthetic Pigments

**Figure 3:**
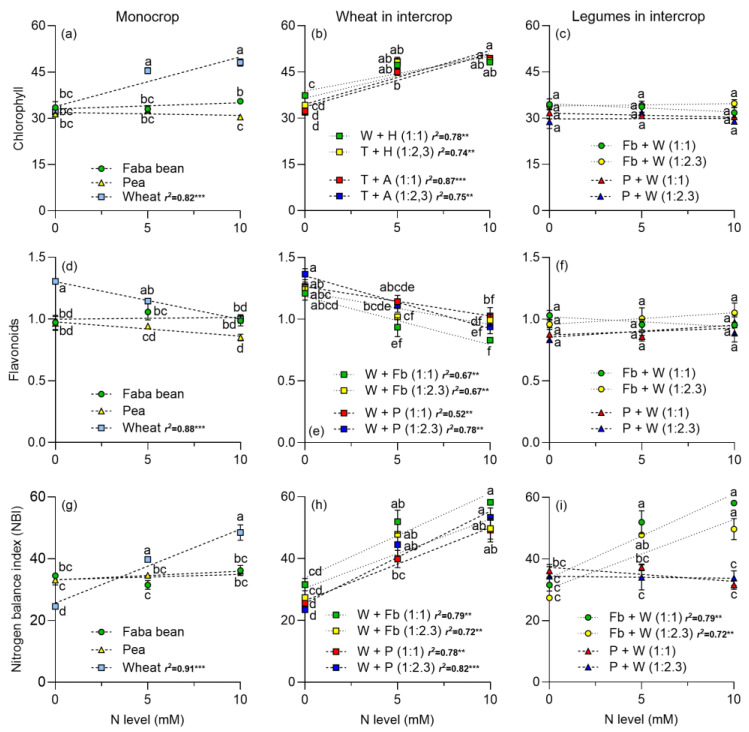
Responses of leaf chlorophyll (**a**–**c**), flavonoids (**d**–**f**) and nitrogen balance index (NBI) (**g**–**i**) at 59 DAS to N level in faba beans, peas, and wheat growing in monocrop and intercropped at two plant densities: 1:1 and 1:2.3, respectively. N levels were zero (N available in the soil), 5, and 10 mM applied in the form of NH_4_NO_3_. Symbols represent the mean and bars the standard error (*n* = 4 for each N level). r^2^ is the coefficient of determination for linear regressions and asterisks indicate statistical significance at *p*-values: ** < 0.001, and *** < 0.0001, respectively. Small letters indicate differences between treatments according to two-way ANOVA and Tukey tests (*p* < 0.05).

Photosynthetic pigments were analyzed at 59, 66, and 74 days after sowing (DASs). Since pigment contents were similar at the three measurement dates, data obtained at 59 DASs are presented in Figure 3 (see Supplemental Material Figures S1 and S2 for pigments at 66 and 74 DAS). For wheat cropped alone or intercropped, the chlorophyll content increased with N level, while for legumes there was no significant effect (Figure 3a–c). Flavonoids decreased in wheat as the N increased, either cropped alone or intercropped with legumes (Figure 3d,e). In addition, N did not alter the flavonoid content in legumes (Figure 3f). When it comes to the nitrogen balance index (NBI), this increased in wheat as the N level increased, either in monocrop or intercropped (Figure 3g,h). However, it remained constant in peas, both in monocrop and intercropped. Interestingly, NBI increased as the N level increased for faba beans intercropped with wheat at both plant densities tested (Figure 3i).

### 2.3. Dry Matter and Land Equivalent Ratio (LER)

**Figure 4:**
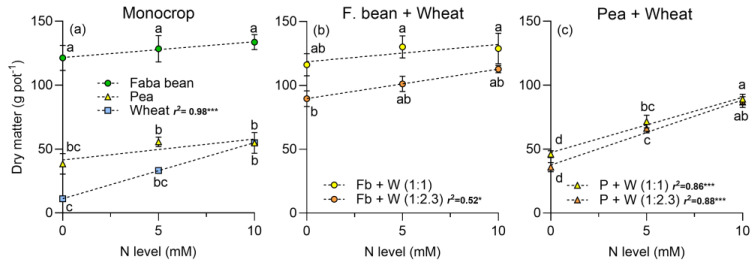
Shoot dry matter per pot in (**a**) faba beans, peas, and wheat in monocrop; (**b**) total dry matter produced by the faba bean–wheat intercrop, and (**c**) total dry matter produced by the pea–wheat intercrop, at two plant densities, 1:1 and 1:2.3 and three N levels, respectively. N levels were 0 (N available in the soil), 5, and 10 mM applied in the form of NH_4_NO_3_. Symbols represent the mean and bars the standard error (*n* = 4 for each N supply). r^2^ is the coefficient of determination for linear regressions and asterisks indicate statistical significance at *p*-values: * < 0.05 and *** < 0.0001, respectively. Small letters indicate differences among treatments according to two-way ANOVA and Tukey tests (*p* < 0.05).

In monocrops, the shoot dry matter (DM) of faba beans was higher than wheat or peas, at any N level (Figure 4a). For the faba bean–wheat intercrop, the shoot DM was higher at the 1:1 plant density compared to the 1:2.3 (Figure 4b); the slope of the relationship between DM yield and N level was significant at the 1:2.3 plant density. For the pea–wheat intercrop, the shoot DM was higher compared to the monocrop, especially at 10 mM of N (Figure 4c). For this combination, the factors plant density, N level, and their interaction were significant (Table 1).

**Figure 5:**
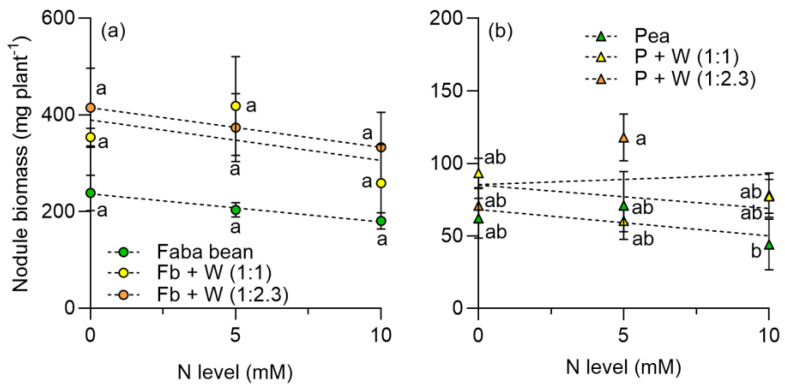
Nodule biomass per plant in (**a**) faba beans in monocrop and intercropped with wheat and (**b**) peas in monocrop and intercropped with wheat at two plant densities, 1:1 and 1:2.3 and at three N levels, respectively. N levels were zero (N available in the soil), 5, and 10 mM applied in the form of NH_4_NO_3_. Symbols represent the mean and bars the standard error (*n* = 4 for each N level). Small letters indicate differences among treatments according to two-way ANOVA and Tukey tests (*p* < 0.05).

For faba beans, the nodule biomass per plant slightly decreased as N increased in monocrop and intercrop with wheat (Figure 5a); the nodule biomass (nodule density) was lower in the monocrop compared to the intercrop. For peas in monocrop, nodule biomass decreased in response to the added N (Figure 5b). However, there was no variation in the nodule biomass across the N levels for peas intercropped with wheat, except for peas in monocrop at the high N dose.

**Figure 6:**
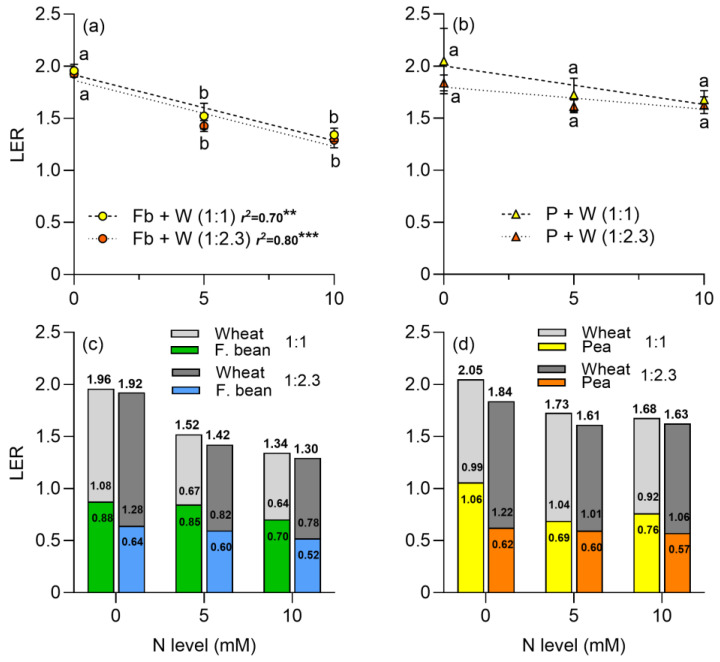
Land equivalent ratio (LER) of (**a**) faba bean–wheat intercrop, (**b**) pea–wheat intercrop, at two plant densities, 1:1 and 1:2.3 and at three N levels. The relative contribution of faba beans and wheat (**c**) and peas and wheat (**d**) in the LER are also shown. N levels were zero (N available in the soil), 5, and 10 mM applied in the form of NH_4_NO_3_. Symbols represent the mean and bars the standard error (*n* = 4 for each N level). In (**a**,**b**), r^2^ is the coefficient of determination for linear regression and asterisks indicate statistical significance at *p*-values: ** < 0.001 and *** < 0.0001, respectively. In (**c**,**d**), the numbers above the bars are the LER and the numbers inside the bars are the relative contribution of wheat and legumes to the LER. Small letters in (**a**,**b**) indicate differences between treatments according to two-way ANOVA and Tukey tests (*p* < 0.05).

The LER decreased as the N level increased for faba beans intercropped with wheat independent of plant density (Figure 6a; Table 1). The relative contribution of wheat was higher when it was intercropped with peas than with faba beans (Figure 6c,d). As the N level increased, the relative contribution of wheat decreased when it was intercropped with faba beans (Figure 6c); although its contribution increased when it was intercropped with peas at 1:1 plant density; otherwise, the wheat contribution was maintained at the different N levels when density increased (Figure 6d).

### 2.4. Proportion of Nitrogen Derived from Air (%Ndfa), N Transfer, and Equivalent N Uptake Ratio (LER_N_)

**Figure 7:**
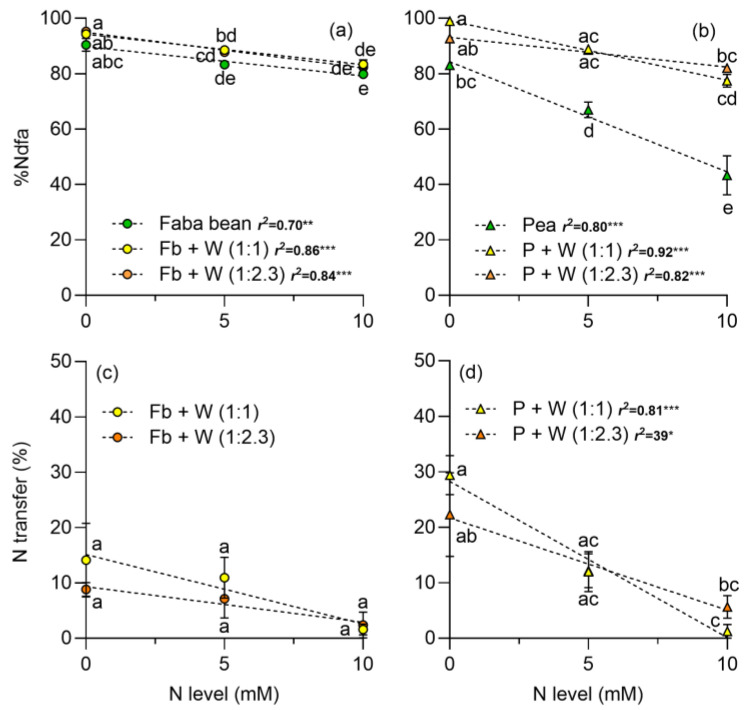
Proportion of N derived from air (%Ndfa) in (**a**) faba beans in monocrop and intercrop with wheat and (**b**) peas in monocrop and intercrop with wheat. The N transferred from (**c**) faba beans to wheat and (**d**) peas to wheat at two plant densities, 1:1 and 1:2.3, and at three N levels, respectively. N levels were zero (N available in the soil), 5, and 10 mM applied in the form of NH_4_NO_3_. Symbols represent the mean and bars the standard error (*n* = 4 for each N level). r^2^ is the coefficient of determination for linear regressions and asterisks indicate statistical significance at *p*-values: * < 0.05, ** < 0.001, and *** < 0.0001, respectively. Small letters indicate differences among treatments according to two-way ANOVA and Tukey tests (*p* < 0.05).

The %Ndfa was above 80% in both legumes when there was no N addition. For faba beans, the %Ndfa decreased as the N level increased, and no differences were observed between intercropped or monocropped treatments (Figure 7a). For peas, the intercropping with wheat reduced the inhibition of %Ndfa due to N addition (Figure 7b). The maximum reduction of the %Ndfa by N was lower in faba beans (12%) than in peas (40%). In the pea–wheat intercrop the %Ndfa at the highest N level was maintained at 77.4 and 82% at 1.1 and 1:2.3 plant density, respectively (Figure 7b). The effects of plant density and N dose were significant in faba beans and peas, and also the plant density × N level interaction was significant for peas (Table 2).

Nitrogen transfer from legumes to wheat was drastically reduced as the N level increased (Figure 7c,d; Table 2). In the faba bean–wheat intercrop at the 1:1 plant density, the N transfer to the wheat was of 14.1%, 10.9%, and 1.6% at 0, 5, and 10 mM of N, respectively. In contrast, at the 1:2.3 plant density, N transfer decreased from 8.8% to 7.2% and 2.4% at the 0, 5, and 10 mM of N rates, respectively (Figure 7c). In the pea–wheat intercrop at the 1:1 plant density there was a maximum transfer of 29.4%, 12.0%, and 1.2% at 0, 5, and 10 mM of N, respectively. At the 1:2.3 plant density, the N transfer decreased from 22.3 to 12.2 and to 5.7% at 0, 5, and 10 mM of N, respectively (Figure 7d).

**Figure 8:**
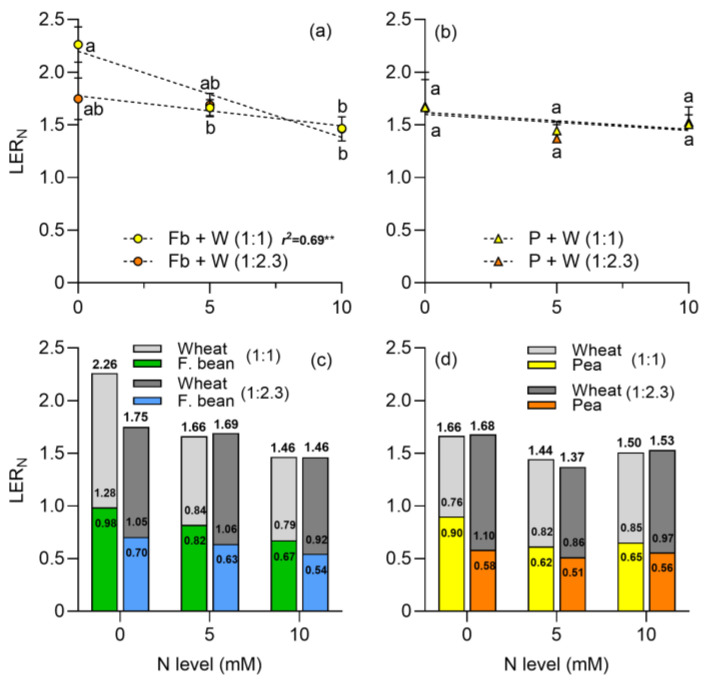
Land equivalent ratio for N (LER_N_) of (**a**) faba bean–wheat intercrop, (**b**) pea–wheat intercrop, at two plant densities, 1:1 and 1:2.3 and at three N levels. The relative contribution of faba beans and wheat (**c**) and peas and wheat (**d**) in the LER_N_ are also shown. N levels were zero (N available in the soil), 5, and 10 mM applied in the form of NH_4_NO_3_. Symbols represent the mean and bar the standard error (*n* = 4 for each N level). In (**a**,**b**), r^2^ is the coefficient of determination for linear regression and asterisks indicate statistical significance at *p*-value lower than ** < 0.001. In (**c**,**d**), the numbers above the bars are the LER_N_ and the numbers inside the bars are the relative contribution of wheat and legumes to the LER_N_. Small letters in (**a**,**b**) indicate differences between treatments according to two-way ANOVA and Tukey tests (*p* < 0.05).

For faba beans, the land equivalent ratio for N uptake (LER_N_) decreased with N supply, especially at the 1:1 plant density (Figure 8; Table 2). At the 1:1 plant density, a maximum value of 2.3 and a minimum of 1.5 were recorded at 0 and 10 mM of N, respectively (Figure 8a). For the pea–wheat intercrop, the LER_N_ values were similar at both plant densities and were not affected by N supply (Figure 8b).

In relation to the relative contribution of each species, in the intercropping of faba beans and wheat, a higher contribution of wheat was observed with respect to faba beans when there was no N addition, especially at the 1:1 plant density (Figure 8c). In the pea–wheat mixture, the lowest N level also presented the highest ratio in favor of peas at the 1:1 plant density (Figure 8d). However, this value also decreased as N increased. On the other hand, at the 1:2.3 plant density, as the N level increased, the relative contribution of wheat underwent less variation than at the 1:1 plant density, particularly in the intercropping of faba beans with wheat (Figure 8c).

## 3. Discussion

### 3.1. Overall Aspects

Intercropping legumes with cereals enhances complementarity processes [7]. The addition of N to the intercropping systems predominantly benefits wheat, fostering increased photosynthesis and the accumulation of photosynthetic pigments in both monoculture or intercropped with *P. sativum* or *V. faba* (Figure 1, Figure 2 and Figure 3). Notably, legumes showed no significant response to N addition, indicating that N_2_ fixation adequately supplied the N required under the experimental conditions. Furthermore, the addition of N boosted DM yield in wheat, and it had no positive effect on legumes (Figure 4). The DM yield is reflected in the LER, which declined as N addition and wheat density increased, with higher values observed in intercrops without N supply (Figure 6). The increased N uptake by wheat helped to reduce the inhibition of N_2_ fixation caused by the N addition, especially of *P. sativum* (Figure 7a,b). Alternatively, legumes use N_2_ fixation for their own metabolism, thereby increasing the availability of N for wheat and the transfer of N compounds via rhizodeposition [6] (Figure 7c,d). The higher %Ndfa found in legumes without N addition, suggests that N_2_ fixation and N transfer to the cereal are more efficient under low N conditions. In the following sections, we will describe these effects in detail.

### 3.2. Leaf Photosynthetic Traits

The increase in leaf gas exchange (A_n_, E and g_s_) of wheat growing in monoculture or intercrop is consistent with the increase observed in the leaf chlorophyll content (Figure 1 and Figure 2). The positive relationship between leaf N or chlorophyll content and A_n_ or g_s_ has been well stablished in different species, including wheat and faba beans [41,42,43]. The higher A_n_ and g_s_ of wheat growing in intercropping compared to monoculture, particularly with no N addition, can be explained by the lower competition for resources in the intercrop. The lack of response of A_n_ and g_s_ of legumes to N addition was probably a consequence of the leaf N status of legumes (NBI), especially for peas which showed no change at the different N levels (Figure 3). This indicates that N_2_ fixation can sustain the growth of legumes without extra N.

Photosynthetic pigments are useful for estimating the level of N in plants and are directly related to biomass production and photosynthesis [44,45]. Other investigations on legumes intercropped with non-legumes have demonstrated an increase in wheat chlorophyll content compared to monoculture, particularly combined with N supplementation, e.g., Tosti and Guiducci [46] in wheat–faba bean; and Suryapani et al. [47] in lentil–wheat. Experiments with soybeans reinforce the idea that intercropping with a legume increases the amount of chlorophyll in both species [39], even if the companion crop is not a cereal, e.g., mint or tea [39,48,49].

The slightly higher NBI of wheat in intercrop compared to being cropped alone can be associated with a higher N availability in soil and the N transferred from the intercropped legumes. Flavonoids exhibit antioxidant characteristics and play a role in safeguarding cells during periods of stress, such as N deficiency. In conditions of N sufficiency, plants prioritize chlorophyll synthesis, whereas during N deficit, they employ flavonoid production as a protective measure [50]. In the three measurements performed on wheat, flavonoid content decreased as the N level increased (Figure 3 and Supplemental Figure S2). This contrasted with the results obtained in legumes, where the concentration of flavonoids did not vary with the amount of N applied. Therefore, our data indicate that cereals have a higher susceptibility to stress when they lack N to meet their demand, leading to higher flavonoid production. In contrast, legumes are less likely to suffer from N deficiency stress because they can meet their requirements through N_2_ fixation.

### 3.3. Dry Matter Production and Land Equivalent Ratio (LER)

The height of the crop causes interspecific competition for light, benefiting if it is taller, in this case, for wheat [51,52]. Furthermore, plants in intercrop have a higher radiation use efficiency compared to those in monocrop [53,54,55]. The lower biomass production of peas intercropped with wheat compared to faba beans in intercrop suggests that the latter has more complementary physiological and morphological characteristics, resulting in a more efficient use of resources. Faba beans having an upright growth habit with branching stems similar to cereals enables them to compete better with wheat than peas, which have prostrate growth [45].

The positive effect of N in wheat shoot DM was probably related with its more extensive root system and greater soil exploration capacity, which allows it to take up more N, mainly during the vegetative stage [16,29,56]. On the other hand, legumes, both in monoculture and intercropping, did not significantly increase DM when N was added. These results suggest that increased competition from cereals in intercropping forces legumes to supply their N_2_ fixation to meet their demand [10,29,40]. Previous studies supported these results, confirming that cereals are strong competitors for soil resources, especially for N in mixtures of cereals and legumes [11,18].

In relation to the LER, various authors have reported LER values higher than one in intercrop of legumes and cereals, including faba beans [14,57,58] and peas [59,60,61]. This indicates a higher resource use efficiency in intercrop systems compared to monocrops. In the present work, LER decreased as the proportion of wheat plants increased in the intercrop, as reported by Dhima et al. [62] for faba beans intercropped with oat. The higher LER value without N addition suggests that the intercropping of legumes and cereals is especially advantageous in low-input agriculture, especially when N availability is lower [63,64,65]. Regarding the relative contribution of the species to the LER, the shoot DM obtained by wheat represents the high proportion of the LER index [10,63]. Conversely, when the intercrop involves herbaceous grass species such as ryegrass, the legume has a higher relative contribution to the LER [66,67]. Therefore, this pot experiment is aligned with the idea that, under N limited conditions, legume–cereal mixtures can be more efficient in terms of equivalent land use.

### 3.4. N_2_ Fixation, N Transfer, and Land Equivalent Ratio for N (LER_N_)

The results revealed that the %Ndfa of faba beans was less affected by N level than peas, when cultivated as monocrops, as reported previously by Guinet et al. [68]. The nodule biomass was much higher in faba beans, in both monocrop and intercrop (Figure 5). A study by Liu et al. [69] showed that the number of nodules in faba beans slightly decreases as the N level increases, reaffirming the idea that intercropping can inhibit the negative effect of N on nodulation; in the case of peas, nodule biomass was not affected by N increase, although the %Ndfa was significantly reduced (Figure 7). This can be explained by a reduction of specific nodule activity which reduces the amount of N fixed by each nodule.

Previous studies indicate that the intercropping of legumes with cereals improves the nodulation capacity, increasing the number and weight of nodules, especially when there is a higher density of cereal in the mixture [70]. Our results show that, for faba beans, the nodule biomass increased in intercropping with wheat plants. Similar results were reported by Li et al. [71], who recorded higher nodule biomass in intercropping than in monoculture for faba beans. In the case of peas, the nodule biomass was similar in the monocrop and intercrop; however, Hu et al. [72] reported higher nodule biomass in relation to the pea cropped alone. The higher nodule biomass in intercropping systems could be related to a decrease in the soil available N due to the cereal N uptake, which results in the production of nodules to compensate for N uptake by the legume.

The intercropping of wheat and faba beans or peas improved the efficiency of N_2_ fixation and attenuated its inhibition to soil N, particularly benefiting the more sensitive species—i.e., peas (Figure 7b). This can be explained by the fact that cereals have a root system with a greater capacity to take up N compared to legumes, presenting a greater interspecific competition and promoting the process of N_2_ fixation by legumes [27,73,74]. Furthermore, the N transferred to wheat was higher when intercropped with peas than faba beans (Figure 7c,d), suggesting that peas have a higher rate of decomposition and renewal of roots and nodules, which upon completing their life cycle releases N compounds that are mineralized by microorganisms, which is known as rhizodeposition [6,75]. The above could also be related to the precocity of peas compared to faba beans, which could also improve N transfer. In addition, the N application significantly decreased N transfer, particularly in peas, which may be due to the inhibition of N_2_ fixation caused by N application [76,77]. Alternatively, it cannot be ruled out that the addition of N led to a dilution of N from rhizodeposition that affects N transfer. Interestingly, when the intercrop has the same plant density of cereals and legumes, the N transfer was higher than with less wheat plants. This suggests a high N availability for wheat due to lower intraspecific competition.

With respect to the LER_N_, the results support previous findings showing values higher than one in cereal and legume intercrops [29,78], indicating higher efficiency of intercropping over monocrop. Furthermore, in the intercrop of faba beans with wheat at a 1:1 plant density, LER_N_ was higher with no N addition. This coincides with the results of Jiao et al. [79] and Wang et al. [77], who pointed out that N supply decreased the LER_N_. This could be explained by the fact that legumes stop N_2_ fixation and increase competition for N uptake, decreasing intercrop efficiency. In addition to the above, Zhu et al. [80] reported that LER_N_ decreased significantly in legume and cereal intercrops when N, P, and water were more available. This indicates that, at lower resource availability, a positive interspecific interaction is promoted, mainly by stimulating N_2_ fixation in the absence of available soil N [80,81]. The results obtained confirm a higher N use efficiency in intercrop systems with legumes and cereals, in contrast to monocrops. This is largely due to the complementary use of the mineral N present in the soil and the N_2_ fixed by legumes [76,78].

## 4. Materials and Methods

### 4.1. Plant Growth Conditions and Experimental Design

The effects of intercropping on the inhibition of N_2_ fixation by exogenous N addition were evaluated in a pot experiment using the faba bean var. ‘Super Agua Dulce’ and the pea var. ‘Utrillo’ intercropped with soft wheat var. ‘Pandora’, at three levels of N. The experiment was carried out using 10 L plastic pots filled with 12 kg of a 1:1 soil–sand mixture based on weight, which were placed in a laterally open greenhouse (only the roof-covered the plants to shield them from the rain) at the Phenomics Center of the Talca University, Chile, during the 2021 season (Supplemental Figure S3). The soil used was an Inceptisol with a pH of 6.3 (pH–soil to water ratio of 1:2.5) and 1.0, 20, and 196 mg kg^−1^ of N, P, and K, respectively, 1.2% of soil organic matter (SOM), and 0.03 dS m^−1^ of electric conductivity.

Before sowing, seeds of the three species were washed with distilled water, selected according to shape and size and germinated in plastic clamshell containers with sterilized vermiculite and subsequently irrigated with 200 mL of distilled water and placed in a growth chamber (Pitec^®^ BIOREF-38L) at 23 °C with a 12 h light/12 h dark photoperiod. After 3–6 days, the most homogeneous seedlings were selected and transplanted at a depth of 3 cm. In all treatments (monoculture and intercrop) ten seedlings were transplanted per pot. In intercrop treatments, seedlings were transplanted at two densities: 1:1 = 5 legumes and 5 cereals, and 1:2.3 = 3 legumes and 7 cereals. After transplanting, seedlings of legumes were inoculated with 4 mL/plant of a stationary *Rhizobium leguminosarum* bv. viciae YEM culture (local strains 373-3007-Su303 for peas; and strain 1400 for faba beans), with an approximate cell density of 10^9^ mL^−1^ to promote nodulation. Treatments were completely randomized with four replicates and three N levels applied as ammonium nitrate (NH_4_NO_3_): 0, 5, and 10 mM. To achieve an optimal P supply, in addition to the P present in the soil-sand mixture (1:1 ratio), 200 mg of P per kg of soil mixture was added in the form of potassium phosphate (K_2_HPO_4_). All the plants were fertilized with a nutrient solution composed of 0.7 mM of K_2_SO_4_, 0.5 mM of MgSO_4_, 0.8 mM of CaCl_2_, 4.0 µM of H_3_BO_3_, 0.1 µM of Na_2_MoO_4_, 1.0 µM of ZnSO_4_, 2.0 µM of MnCl_2_, 0.2 µM of CoCl_2_, 1.0 µM of CuCl_2_, and 1.0 µM of FeNaEDTA. The nutrient solution was applied at a frequency of every four days, with each application consisting of a volume of 500 mL. In the case of N, it was applied alongside the nutrient solution according to the respective treatment, 0, 5, or 10 mM, but only once a week and starting three weeks after transplanting. The soil water content was maintained at 75% of the maximum water-holding capacity.

The experimental design considered two factors: the N dose and plant density in the intercropping system, using four replicates for all treatments, except for the treatment of faba beans intercropped with wheat at a plant density of 1:1 and 10 mM of N, where 3 replicates were recorded. Analyses of variance (two-way ANOVA) were performed for all measured variables after testing for normality and homogeneity of variances using Shapiro–Wilk and Levene’s tests, respectively. Plots, regressions, and their parameters were calculated using GraphPad Prism^®^ version 10.0.

### 4.2. Gas Exchange and Photosynthetic Pigment Content

Leaf gas exchange was determined using a CIRAS-2 gas analyzer. Measurements were taken between 11:00 and 14:00 h, during the peak of photosynthetic activity. The parameters evaluated were net assimilation of CO_2_ (A_n_), transpiration (E), stomatal conductance (g_s_), intercellular CO_2_ concentration (C_i_), and water use efficiency (WUE), the latter calculated as a ratio of A_n_–E. The conditions under which this measurement was carried out were with an intensity of photosynthetic active radiation (PAR) from 1500 to 2100 µmol m^−2^s^−1^, leaf temperature between 23 and 25 °C, and an airflow rate of 248 mL/min. In the case of the legumes, measurements were made in the middle of the plant, considering the central part of a leaf in good condition. For the wheat, measurements were made on the flag leaf. Measurements were taken 63 days after sowing (DAS), with the legumes at an advanced stage of flowering and the wheat at boot stage. The parameters were measured three times in each leaf and in triplicate for each treatment.

The content of photosynthetic pigments (chlorophyll, flavonoids, and the nitrogen balance index [NBI]) was determined using a portable chlorophyll meter (DUALEX^®^) on the same leaves used previously for the measurement of gas exchange, but three measurements were made on different dates. The first measurement was taken at 59 DAS, at which time the legumes were in full flowering and the wheat was in the boot stage. The second measurement was taken at 66 DAS when the legumes were in pod formation and the wheat was at anthesis. Finally, the third measurement was made at 74 DAS when the legumes and wheat were in the grain-filling stage. Three plants per treatment, one leaf per plant, and three measurements on the same leaf were evaluated.

### 4.3. Determination of Plant and Nodule Dry Matter

The dry matter (DM) of shoots and nodules was evaluated at the grain filling stage. All plants were harvested at 77 DASs. Shoots were separated from the roots and dried in an air oven at 65 °C for 48 h until reaching a constant weight. The roots were carefully separated from the soil, washed, and stored at −80 °C. Then, the nodules were separated from the roots manually with steel tweezers. Subsequently, the nodules were dried at 65 °C for 48 h for DM determination. Shoot DM was finely milled and stored in paper bags for subsequent chemical analysis.

### 4.4. Determining the Proportion of N Derived from Air (%Ndfa)

The N concentration and ^15^N natural abundance (expressed as ‰ δ^15^N relative to the ^15^N composition of atmospheric N_2_) were determined using an elemental analyzer and an isotope ratio mass spectrometer at the Laboratory of Applied Chemistry and Physics, Ghent University, Belgium. The natural abundance method of ^15^N, to measure the fixation of N_2_, is based on the difference that exists between the abundance of ^15^N from atmospheric N_2_ (δ^15^N = 0‰) and the N present in the soil (δ^15^N generally > 0‰) [82]. The percentage of N derived from the air (%Ndfa) in legumes was calculated by comparing natural abundance of ^15^N of the legume (δ^15^N_leg_) with that of the reference plants (δ^15^N_ref_), using Equation (1) [83]:

(1) $$\%Ndfa=100\times \left[\frac{\delta^{15}N_{ref}-\delta^{15}N_{leg}}{\delta^{15}N_{ref}-\beta }\right]$$

where, the reference plant provides the natural abundance of ^15^N that comes from the soil and the β value represents the ^15^N abundance in legumes that rely solely on N_2_ fixation for growth [82]. With this method, the greater the difference in the natural abundance of ^15^N between the reference plants and the legumes, the greater the N_2_ fixation, and the closer the δ^15^N_leg_ is to the β value. The reference plants used were wheat (*Triticum aestivum* L.), oat (*Avena sativa* L.), sunflower (*Helianthus annuus* L.), and quinoa (*Chenopodium quinoa* Willd.). These plants were grown under the same experimental conditions as the legumes, and with the addition of 5 mM of N. The shoots of the reference plants were harvested, dried, weighed, and milled in the same way as described above for the legumes. The reference plants were harvested manually at the phenological stage of anthesis for wheat and oat or at full flowering for quinoa and sunflower.

### 4.5. Determination of the β Value

Legumes have different N_2_ fixing capacities, which is reflected in the β value, which indicates the amount of N_2_ fixed in the absence of N in the substrate [82,83]. Because the β value was not determined in the absence of N, a sensitivity analysis was performed, with a range of β values obtained from the literature and serving to evaluate the effect of N on the inhibition of N_2_ fixation (Supplemental Figure S4).

### 4.6. N Transfer from Legume to Cereal

To measure N transfer from legumes to cereals, the δ^15^N present in the cereal in monocrop was compared with the δ^15^N of the cereal intercropped with the legumes. The %Ndfa transferred from legume to cereal (%N_t_) was calculated by Equation (2):

(2) $${\%N}_{t}=100\times \left[1-\frac{\delta^{15}N_{Cereal\;IC}}{\delta^{15}N_{Cereal\;MC}}\right]$$

where δ^15^N_Cereal IC_ represents the δ^15^N in the cereal intercropped with legumes and δ^15^N_Cereal MC_ in the monocrop.

### 4.7. Land Equivalent Ratio (LER)

To calculate the land use efficiency, the land equivalent ratio (LER) was used, which compares the yield achieved by intercropping two species with the yield obtained in a monocrop. The LER was calculated according to Equation (3) proposed by Mead and Willey [34]:

LER = (SB_Leg IC_/SB_Leg MC_) + (SB_Cereal IC_/SB_Cereal MC_) (3)

where, SB_Leg IC_ is the legume shoot biomass in intercropping, SB_Leg MC_ is the legume shoot biomass in monocrop, SB_Cereal IC_ is the cereal shoot biomass in intercropping and SB_Cereal MC_ is the cereal shoot biomass in monocrop. In addition, to calculate the equivalent N uptake ratio (LER_N_), the same Equation (3) was used, but with the values of N content accumulated in the shoot.

## 5. Conclusions

The intercrop of legumes with wheat can improve N use efficiency, since cereals, being more competitive, decrease the inhibition of N_2_ fixation caused by the presence of N in the soil. In addition, peas were found to be more sensitive to the presence of N than faba beans, so the latter could have a better complementarity with wheat. However, peas can transfer up to 30% of the N fixed, so this is also a species that should be considered in intercropping systems. In addition, when there is a lower amount of N in the soil, N_2_ fixation and N transfer are promoted, which directly benefits the performance of wheat, increasing its N content, photosynthetic parameters, and LER and LER_N_ indexes.

Therefore, this study provides a deeper understanding of the dynamics in legume–cereal intercropping. The role of N in competition, N fixation and transfer, and photosynthesis, highlighting the complexity of interspecific interactions and how they can influence agricultural productivity and the efficient use of N. These findings have important implications for the planning of sustainable cropping systems and the optimization of food production.

## Figures and Tables

**Table 1:** Two-way ANOVA results for the effects of plant density, N dose, and their interactions in DM produced per area, DM of nodules, and LER.

Factors	Variable	*df*	F-Value	*p*-Value
*A*: Plant density	*V. faba* shoot DM	2	10.20	<0.001 ***
*B*: N dose		2	3.11	0.06
*A × B*		4	0.23	0.92
*A*: Plant density	*P. sativum* shoot DM	2	11.17	<0.001 ***
*B*: N dose		2	40.36	<0.001 ***
*A × B*		4	3.23	0.03 *
*A*: Plant density	*V. faba* nodule DM	2	6.19	0.006 **
*B*: N dose		2	1.44	0.25
*A × B*		4	0.39	0.81
*A*: Plant density	*P. sativum* nodule DM	2	3.08	0.12
*B*: N dose		2	0.95	0.40
*A × B*		4	2.03	0.12
*A*: Plant density	LER for *V. faba*	1	0.94	0.35
*B*: N dose		2	38.3	<0.001 ***
*A × B*		2	0.09	0.91
*A*: Plant density	LER for *P. sativum*	1	0.93	0.35
*B*: N dose		2	2.16	0.14
*A × B*		2	0.12	0.88

Asterisks indicate statistical significance at * < 0.05, ** < 0.01, and *** < 0.001.

**Table 2:** Two-way ANOVA results for the effects of plant density, N dose, and their interactions in %Ndfa, %N transferred, and LER_N_ in different crop treatments.

Factors	Variable	*df*	F-Value	*p*-Value
*A*: Plant density	%Ndfa *V. faba*	2	9.96	<0.001 ***
*B*: N level		2	57.5	<0.001 ***
*A* × *B*		4	0.32	0.86
*A*: Plant density	%Ndfa *P. sativum*	2	72.3	<0.00 ***
*B*: N level		2	56.3	<0.001 ***
*A* × *B*		4	7.07	<0.001 ***
*A*: Plant density	%N transferred *V. faba*	1	0.87	0.36
*B*: N level		2	3.74	0.04 *
*A* × *B*		4	0.38	0.68
*A*: Plant density	%N transferred *P. sativum*	1	0.06	0.80
*B*: N level		2	15.8	<0.001 ***
*A* × *B*		4	1.05	0.37
*A*: Plant density	LER_N_ *V. faba*	1	2.22	0.15
*B*: N level		2	8.02	0.004 **
*A* × *B*		2	2.60	0.10
*A*: Plant density	LER_N_ *P. sativum*	1	0.008	0.93
*B*: N level		2	0.93	0.41
*A* × *B*		2	0.03	0.96

Asterisks indicate statistical significance at *p* * < 0.05, ** < 0.01, and *** < 0.001.

## Data Availability

The data presented in this study are available on request from the corresponding author.

## Supplementary Materials

The following supporting information can be downloaded at: https://www.mdpi.com/article/10.3390/plants13070991/s1, Figure S1: Responses of leaf chlorophyll (a–c), flavonoids (d–f), and nitrogen balance index (NBI) (g–i) at 66 DAS to N level in faba beans, peas, and wheat grown in monocrop and intercropped at two plant densities: 1:1 and 1:2.3, respectively. N levels were zero (N available in the soil), 5, and 10 mM applied in the form of NH_4_NO_3_. Symbols represent the mean and bars the standard error (*n* = 4 for each N level). r^2^ is the coefficient of determination for linear regressions and asterisks indicate statistical significance at *p*-values: * < 0.05, ** < 0.001, and *** < 0.0001, respectively. Small letters indicate differences between treatments according to two-way ANOVA and Tukey tests (*p* < 0.05); Figure S2: Responses of leaf chlorophyll (a–c), flavonoids (d–f), and the nitrogen balance index (NBI) (g–i) at 74 DAS to N level in faba beans, peas, and wheat grown in monocrop and intercropped at two plant densities: 1:1 and 1:2.3, respectively. N levels were zero (N available in the soil), 5, and 10 mM applied in the form of NH_4_NO_3_. Symbols represent the mean and bars the standard error (*n* = 4 for each N level). r^2^ is the coefficient of determination for linear regressions and asterisks indicate statistical significance at *p*-values: * < 0.05, ** < 0.001, and *** < 0.0001, respectively. Small letters indicate differences between treatments according to two-way ANOVA and Tukey tests (*p* < 0.05); Figure S3. Daily mean air temperature, air humidity, and hours of daylight (photoperiod [P]) during the growth period of faba beans, peas, and wheat grown in monocrop and intercropped at two plant densities: 1:1 and 1:2.3, respectively. Plants were grown during the springtime in the southern hemisphere (September to November 2021); Figure S4. Sensitivity analysis for β values for *V. faba* (a–c) and *P. sativum* (d–f) in monocrop and intercropped with *T. aestivum* at two plant densities (1:1 and 1:2.3) and three N levels, respectively. N levels were zero (N available in the soil), 5, and 10 mM applied in the form of NH_4_NO_3_. A range of β values were tested as recorded in the literature. The β values resulted in an average of −0.49 and −0.61, for peas and faba beans, respectively. In the case of faba beans, the β value proposed by the literature correctly fitted the linear regression (the green line in Figure S4a–c), since it never exceeded 100% of %Ndfa. However, the β value used for the peas did not fit well, thus a range of β values were analyzed in a sensitivity analysis and the one that best fitted the linear regression was β = −1.3 (the green line in Figure S4d–f). Independently of the β value used, it was observed that the %Ndfa decreases as N increases.
