# Supplementary material for: N2 Fixation, N Transfer, and Land Equivalent Ratio (LER) in Grain Legume–Wheat Intercropping: Impact of N Supply and Plant Density
Source: Plants (Basel). 2024 Mar 30;13(7):991. doi: 10.3390/plants13070991 (PMC11013795; doi:10.3390/plants13070991)
Supplement: Supplementary file 1 [file plants-13-00991-s001.zip › plants-2914533-supplementary.pdf]

Supplementary materials

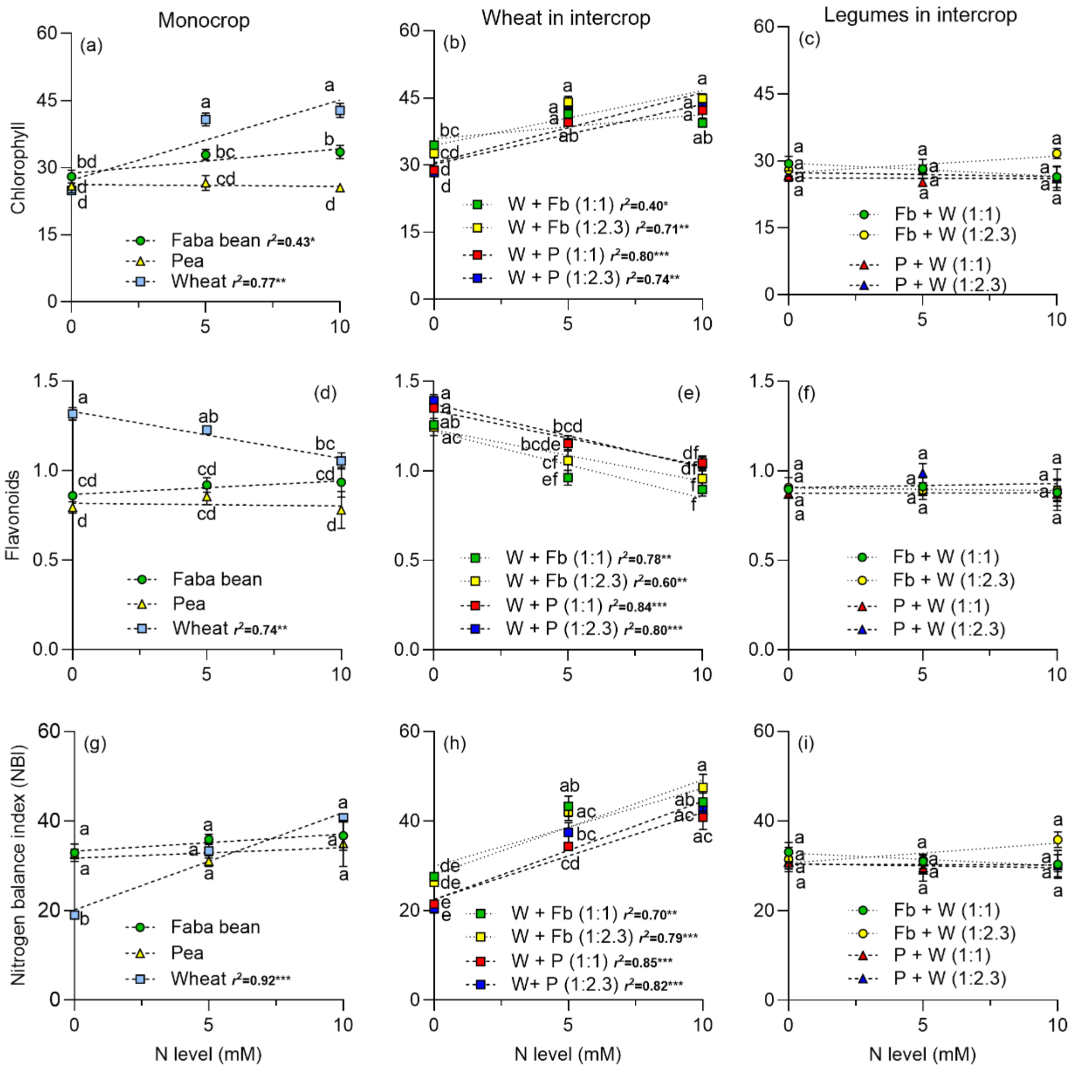

Figure S1. Responses of leaf chlorophyll (a–c), flavonoids (d–f), and nitrogen balance index (NBI) (g–i) at 66 DAS to N level in faba beans, peas, and wheat grown in monocrop and intercropped at two plant densities: 1:1 and 1:2.3, respectively. N levels were zero (N available in the soil), 5, and 10 mM applied in the form of NH_4NO_3. Symbols represent the mean and bars the standard error (n = 4 for each N level). r^2 is the coefficient of determination for linear regressions and asterisks indicate statistical significance at p-values: * <0.05, ** <0.001, and *** <0.0001, respectively. Small letters indicate differences between treatments according to two-way ANOVA and Tukey tests ($p < 0.05$).

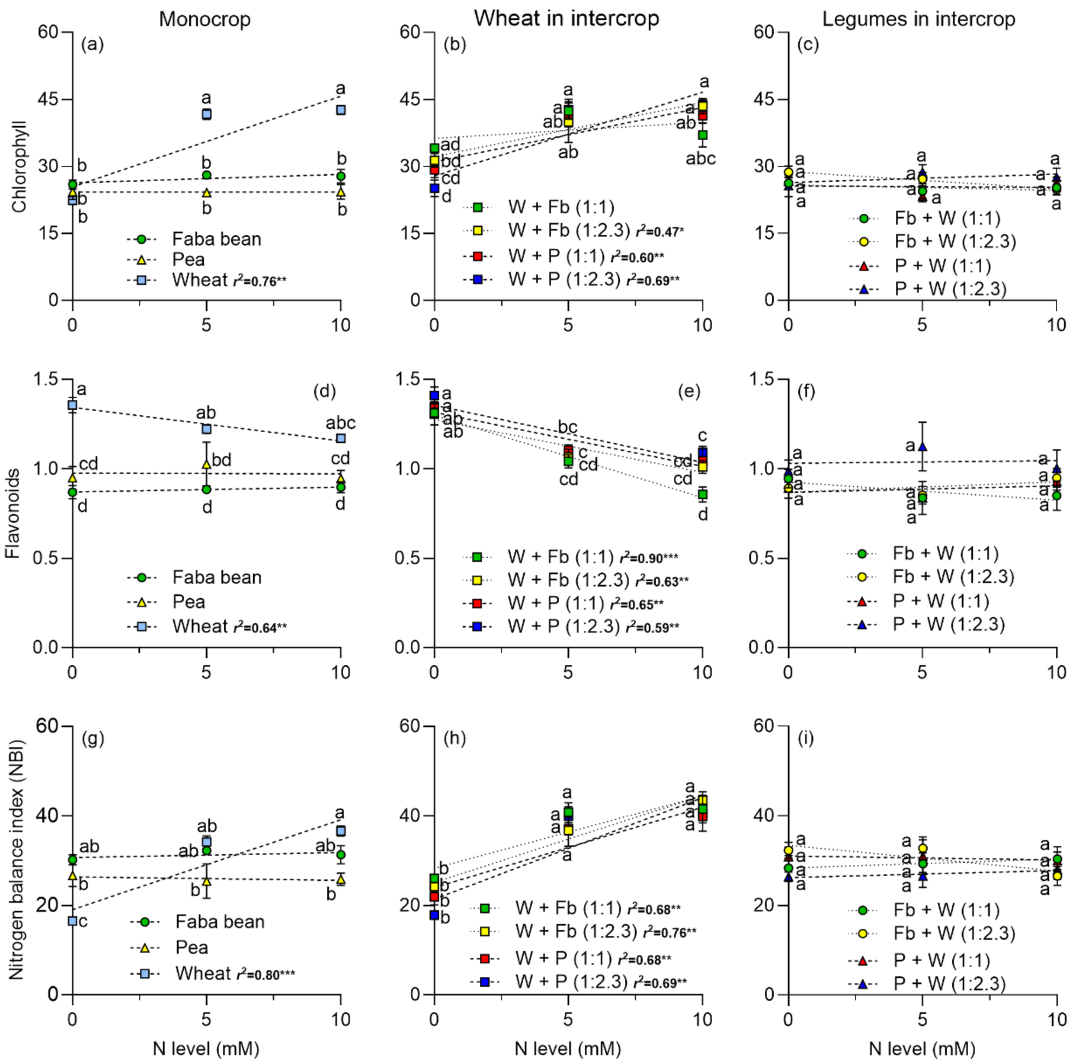

Figure S2. Responses of leaf chlorophyll (a–c), flavonoids (d–f), and the nitrogen balance index (NBI) (g–i) at 74 DAS to N level in faba beans, peas, and wheat grown in monocrop and intercropped at two plant densities: 1:1 and 1:2.3, respectively. N levels were zero (N available in the soil), 5, and 10 mM applied in the form of NH_4NO_3. Symbols represent the mean and bars the standard error (n = 4 for each N level). r^2 is the coefficient of determination for linear regressions and asterisks indicate statistical significance at p-values: * <0.05, ** <0.001, and *** <0.0001, respectively. Small letters indicate differences between treatments according to two-way ANOVA and Tukey tests (p < 0.05).

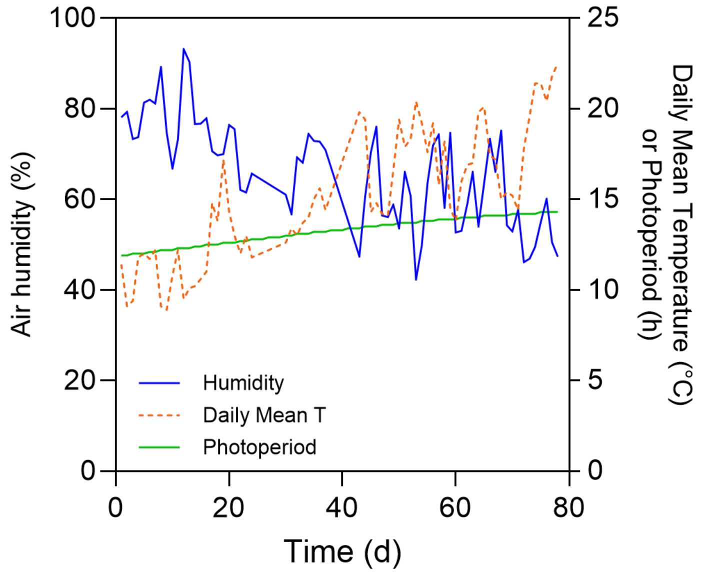

Figure S3. Daily mean air temperature, air humidity, and hours of daylight (photoperiod [P]) during the growth period of faba beans, peas, and wheat grown in monocrop and intercropped at two plant densities: 1:1 and 1:2.3, respectively. Plants were grown during the springtime in the southern hemisphere (September to November 2021).

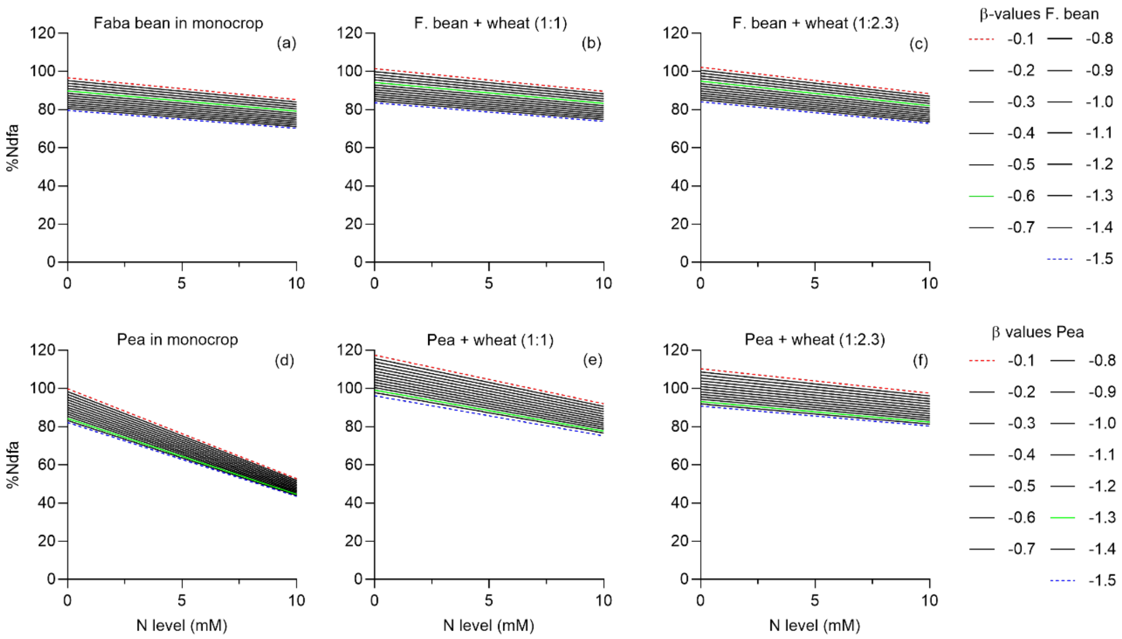

Figure S4. Sensitivity analysis for β values for *V. faba* (a–c) and *P. sativum* (d–f) in monocrop and intercropped with T. aestivum at two plant densities (1:1 and 1:2.3) and three N levels, respectively. N levels were zero (N available in the soil), 5, and 10 mM applied in the form of NH_4NO_3. A range of β values were tested as recorded in the literature. The β values resulted in an average of −0.49 and −0.61, for peas and faba beans, respectively. In the case of faba beans, the β value proposed by the literature correctly fitted the linear regression (the green line in Figure S4a, b, and c), since it never exceeded 100% of %Ndfa. However, the β value used for the peas did not fit well, thus a range of β values were analyzed in a sensitivity analysis and the one that best fitted the linear regression was β = −1.3 (the green line in Figure S4d, e, and f). Independently of the β value used, it was observed that the %Ndfa decreases as N increases.
